# Measuring what matters: Modern evaluation practices in clinical and translational science

**DOI:** 10.1017/cts.2026.10739

**Published:** 2026-04-13

**Authors:** Boris Volkov, Valerie Harder, Brenda Joly

**Affiliations:** 1 Clinical and Translational Science Institute and Institute for Health Informatics, University of Minnesota, Minneapolis, MN, USA; 2 Departments of Pediatrics and Psychiatry, The Robert Larner, M.D. College of Medicine, University of Vermont, Burlington, VT, USA; 3 Public Health Program and Northern New England Clinical and Translational Research Network, University of Southern Maine, Portland, ME, USA

**Keywords:** Evaluation, clinical and translational science, capacity building, continuous improvement, impact

Evaluation is central to the clinical and translational science enterprise, but what counts as “good evaluation” is being fundamentally reimagined. The Clinical and Translational Science Award (CTSA) Program [1] and the IDeA Clinical and Translational Research (CTR) Program [2] communities are evolving from compliance-oriented tracking and narrow academic metrics toward innovative and evidence-informed approaches that contribute to the science of evaluation. This special issue, “Advancing Evaluation Practices in Clinical and Translational Science/Research,” showcases that evolution by highlighting novel advancements, methodologies, and transformative practices in the evaluation of clinical and translational science. Collectively, the articles move the field toward more rigorous, use-driven evaluation that is highly attentive to impact. We hope this special issue generates new ideas, encourages innovation, and continues to advance the discipline through methodologically sound, creative, and effective approaches.

The articles in this issue cluster into five themes. The first, *Reframing Translational Impact*, includes articles advancing the use of the Translational Science Benefit Model by focusing on impact methodology [3], return on investment and benefits of seed funding [4], and community engagement and impact [5]. Also grouped in this theme is an article exploring a conceptual model for translational science impact [6]. The second cluster, *Using Innovative Measurement Tools*, brings readers’ attention to a novel, comprehensive tool assessing our clinical and translational research workforce [7], a digital tool to increase evaluation capacity [8], and community-engaged development of a mixed-method evaluation tool [9]. The third group, *Building Infrastructures and Cultures for Continuous Improvement*, highlights continuous quality improvement around data infrastructure [10, 11], reimagining evaluation as a catalyst for continuous improvement [12], and cross-institution collaboration [13]. Within this theme, other articles introduce a protocol for tracking scholarly products [14], support partnerships between translational and social science [15], and integrate perspectives and methods from other disciplines [16]. In the fourth theme, *Innovating Methods and Tools for Community Engagement and Inclusiveness*, investigators share useful community engagement evaluation approaches [17] and metrics for measuring engagement [18]. Other studies focus on returning value to participants via a modular digital system [19], applying a structured framework to evaluating community engagement efforts [20], and promoting a data-driven approach to evaluating equity and inclusiveness [21]. The fifth thematic cluster, *Strengthening Evaluation Capacity and Fidelity*, contains studies that improve assessment of fidelity of intervention delivery [22], use meta-evaluation [23], and focus on team science evaluation [24]. Finally, a special communication, reporting on the 2025 Association for Clinical and Translational Science Evaluation Special Interest Group meeting, synthesizes current priorities and innovations emerging from the broader clinical and translational science evaluation community. Taken together with the other contributions, it signals a maturing field that is increasingly deliberate about its own learning agenda [25].

As co-editors of this special issue, we are proud to share these innovative contributions to our field of evaluation, and we would like to thank the authors, peer reviewers, and the Journal of Clinical and Translational Science Editorial Board for providing the forum and support for this important dialogue on evaluation. Our goal was to have translational scientists, clinical researchers, evaluation practitioners, community partners, and CTSA/CTR program stakeholders contribute original articles, systematic reviews, case studies, and other manuscripts addressing critical aspects of evaluation in the field, and we feel we attained this goal with the depth and breadth of articles. We hope that by highlighting innovative evaluation approaches and evaluation capacity building initiatives, identifying evaluation barriers and facilitators, and showcasing real-world evaluation use and impact, this issue will support further evidence-based advances in the field of clinical and translational science. We invite you to explore this issue as we consider what new frontiers in evaluation of clinical and translational science may emerge.
